# Evaluation of T1/T2 ratios in a pilot study as a potential biomarker of biopsy: proven benign and malignant breast lesions in correlation with histopathological disease stage

**DOI:** 10.4155/fsoa-2016-0063

**Published:** 2017-05-02

**Authors:** Marina A Malikova, Jaroslaw N Tkacz, Priscilla J Slanetz, Chao-Yu Guo, Adam Aakil, Hernan Jara

**Affiliations:** 1 Department of Surgery, Boston University, Boston Medical Center, 88 East Newton Street, Collamore Building, Suite D507a, Boston, MA 02118, USA; 2 Department of Radiology, Boston Medical Center, Boston University, Boston, MA 02118, USA; 3 Department of Radiology, Beth Israel Deaconess Medical Center, Harvard Medical School, Boston, MA 02215, USA; 4 Department of Biostatistics, Boston University, Boston, MA 02118, USA

**Keywords:** breast imaging, development of potential biomarker for evaluation of breast disease, magnetic resonance imaging, quantitative magnetic resonance imaging

## Abstract

**Aim:**

Early breast cancer detection is important for intervention and prognosis. Advances in treatment and outcome require diagnostic tools with highly positive predictive value.

**Purpose:**

To study the potential role of quantitative MRI (qMRI) using T1/T2 ratios to differentiate benign from malignant breast lesions.

**Methods:**

A cross-sectional study of 69 women with 69 known or suspicious breast lesions were scanned with mixed-turbo spin echo pulse sequence. Patients were grouped according to histopathological assessment of disease stage: untreated malignant tumor, treated malignancy and benign disease.

**Results & Discussion:**

Elevated T1/T2 means were observed for biopsy-proven malignant lesions and for malignant lesions treated prior to qMRI with chemotherapy and/or radiation, as compared with benign lesions. The qMRI-obtained T1/T2 ratios correlated with histopathology. Analysis revealed correlation between elevated T1/T2 ratio and disease stage. This could provide valuable complementary information on tissue properties as an additional diagnostic tool.

## Lay abstract

Early detection is important for successful intervention in breast cancer. We studied the potential role of quantitative MRI (qMRI) using T1/T2 ratios to differentiate benign from malignant breast lesions. Sixty nine women with breast lesions were scanned with qMRI. Elevated ratios were observed for biopsy-proven malignant lesions and for malignant lesions that were treated prior to qMRI with chemotherapy and/or radiation, as compared with benign lesions. With further studies, this approach could provide valuable information concerning tissue properties in addition to established breast imaging sequences and be an additional diagnostic tool.

## Background

Breast cancer is the most common cancer among women, except for skin cancers [1]. About one in eight (12%) women in the USA will develop invasive breast cancer during their lifetime [1]. According to American Cancer Society, about 246,660 new cases of invasive breast cancer were diagnosed in women in 2016, and 61,000 new cases of carcinoma *in situ* will be diagnosed (carcinoma *in situ* is noninvasive and is the earliest form of breast cancer [1].

Early detection is important for successful intervention and improved prognosis. Advances in the treatment and outcome of patients with breast cancer require the development of diagnostic tools with highly positive predictive value.

Imaging modalities such as mammography, ultrasonography and MRI are routinely used to identify and characterize breast lesions [2,3]. Mammography, for all its limits, is still the only test proven to decrease mortality in multiple, randomized, controlled trials and through experience with population-based screening [4].

There are known limitations of mammography, especially in women with extremely dense breast tissue and inconsistent results when this technique is utilized in detecting small cancers before they are palpable that can improve survival, as well as treatment options [5].

MRI is recognized as a supplement to mammography in the visualization and characterization of breast lesions and over the last decade, MRI was widely adopted for assessment of breast disease [6–9].

Kuhl summarized current guidelines of breast MRI in 2007 review paper and emphasized that compared with mammography and breast ultrasonography, contrast-enhanced MRI is a breast imaging technique that offers not only information on lesion cross-sectional morphology, but also adds information on functional lesion features such as tissue perfusion and enhancement kinetics [10]. Kuhl *et al.* refined management recommendations for women with increased familial risk of breast cancer in a prospective multicenter cohort study based on utilization of MRI [11].

Breast MRI still remains an active research field [12–16]. Although the gamut of MRI techniques for breast cancer diagnosis reported in the scientific literature is very rich, surprisingly; quantitative MRI (qMRI) relaxometry of breast lesions has received much less attention. Early breast MRI work by Medina *et al.* showed that calculated values of the ratio T1/T2 provided improved discrimination of fibroadenomas from adenocarcinomas (p < 0.05) [17]. This ratio has also been found useful for discriminating benign from malignant liver lesions [18].

The need for establishing imaging biomarkers of breast cancer conducive to reducing the need for unnecessary biopsies cannot be overstated. Discriminating breast malignancy by measuring the ratio of the longitudinal to the transverse MRI relation times constitutes a noninvasive approach that is novel to our knowledge; reports in the literature on this subject are nonexistent.

The purpose of this work was to measure the T1/T2 ratios of a variety of breast lesions and to correlate with histopathological findings with the goal of increasing the diagnostic power of MRI.

## Materials & methods

### Study design

This was a cross-sectional study in which any patients scheduled to undergo MRI were asked to undergo an additional imaging sequence (mixed turbo spin echo (mixed-TSE)) following the acquisition of the standard conventional imaging. There was no randomization applied. This study was approved by Boston University Institutional Review Board. Informed consent was obtained from each patient prior to any study procedures being performed in compliance with Declaration of Helsinki.

### Study population

Eighty-four women of different ethnicities with known lesions who were scheduled for breast MRI at Boston Medical Center Radiology Department and met the eligibility criteria listed below were invited to participate in this study. Inclusion criteria called for any woman older than 18 years of age at the time of consent who was scheduled to undergo a breast MRI for a known breast cancer or suspicious breast lesions. Patients who were pregnant or lactating were excluded from participation as the accompanying hormonal changes might confound the ability to accurately detect malignancy [19]. Other exclusion criteria included claustrophobia, presence of metallic foreign body or orbital metal, prior reaction or allergy to intravenous gadolinium, renal insufficiency or failure, renal transplant within the past 3 months and inability to provide written informed consent. Consent was obtained by the clinical research associate for the extra sequence, which was performed at the end of the routine MRI examinations.

Out of the total 84 women scheduled for breast MRI in the study time frame, who initially consented for this study, 69 subjects with 69 known lesions successfully completed all study procedures (82.14% participation rate). Fifteen women did not participate due to the following reasons: four patients changed their mind and withdrew consent after it was initially obtained, five cancellations for scheduled conventional breast MRI occurred, two declined to undergo additional research qMRI sequence after completing routine care sequence, three patients were uncomfortable laying down in the scanner and one developed a headache.

Our primary objective was to assess whether there is a correlation between T1/T2 ratio obtained by qMRI and the assigned level of suspicion based on the clinical diagnosis. Results were correlated with histopathology and clinical staging (stage 0, 1, 2, 3 or 4 or benign). The study cohort was divided into three groups as follows:

- Group 1: primary cancer as defined by no prior treatment based on medical history and cancer diagnosis confirmed by histopathology analysis.
- Group 2: cancer post treatment as defined by any previously treated cancer patient who has undergone therapy with radiation, chemotherapy or some combination.
- Group 3: benign as defined by no histopathologic evidence of malignancy.

The secondary objective was to compare findings of conventional MRI to qMRI results.

Early rapid enhancement after gadolinium administration on conventional MRI was examined in correlation with T1/T2 ratio obtained by qMRI in order investigate their potential complementary use.

### Standard protocol including injection

Images were acquired with a 1.5-T superconducting MRI scanner (Achieva Philips Medical System, Cleveland, OH, USA) and 7-channel breast array (Invivo, FL, USA). Our routine breast imaging protocol includes the following imaging sequences: axial T1 TSE, axial STIR and axial dynamic T1 high resolution isotropic volumetric excitation sequences of both breasts. The volumetric sequences were optimized for a temporal resolution of between 60 and 75 s. The dynamic images were acquired immediately after the intravenous administration of gadopentetate dimeglumine (Magnevist, 0.2 ml/kg, Bayer Healthcare, NJ, USA) and intravenous saline flush (20 cc, rate 2 cc/s). At least four time points were obtained in the dynamic series for kinetic analysis using a CADstream (Merge Healthcare, IL, USA) system.

### qMRI research sequence

Mixed turbo spin echo is a multislice 2D pulse sequence that was applied in the coronal plane providing full bilateral breast coverage with null interslice gap: 0.82 × 0.82 × 3 mm^3^ voxel size. Mixed-TSE combines the principles of T1-weighting by inversion recovery and T2-weighting by dual-TSE sampling into a single mixed MRI acquisition. The mixed-TSE pulse sequence has two echo times (TEs) = 7.1/100 ms, two inversion times (TIs) = 700/5033 ms, an echo train length (ETL) = 18 and repetition time (TR) = 10,066 ms. As per protocol this research scan was obtained 5–6 min post-gadolinium injection. The length of qMRI sequence can vary between 5 and 12 min due to amount of time needed to scan the breast tissue which can vary slightly due to actual difference in breast size of individual subjects.

### Image processing & data analysis

Images were Digital Imaging and Communications in Medicine (DICOM) transferred to our image processing laboratory and prepared for further processing in MathCAD (PTC, MA, USA). Self-coregistered T1 and T2 maps were generated with formulas as described in prior work [20] and used as input for a T1/T2 ratio algorithm that avoids singularities with a pixel-wise Boolean conditional statement. For all the subjects, the lesions were identified by an experienced radiologist, with 7 years of experience in breast imaging, which had access only to conventional MRI data and was blinded to histopathology findings. In every case, rectangular regions of interest (ROI) were placed within the lesion, as identified by the radiologist. Glandular tissues were analyzed bilaterally by qMRI as an internal control for each subject to verify that changes seen in ROIs on qMRI are attributed to the disease process occurring in the breast tissues analyzed. Contralateral breasts were assessed by qMRI to examine bilateral involvement.

The person performing qMRI analysis was blinded to histopathology and had no access to medical records in an effort to avoid selection and measurement bias. Pathology results (stage and/or grade of tumor) were obtained for each subject based on available data including prior surgery or image-guided biopsy, allowing correlation between pathology and qMRI data.

Demographic information, current and past hormonal use, personal history of breast cancer, family history of cancer, menopausal status and day of menstrual cycle were also collected based on comprehensive review of medical records and patient interviews.

### Statistical data analysis

The data obtained from 41 subjects with 46 lesions were analyzed using SAS program, version V 9.1 (SAS Institute Inc., NC, USA). In order to meet our primary objective, the nonparametric analysis was used and correlation between T1/T2 ratios and the grade assigned based on clinical diagnosis was examined.

We performed stratified data analysis and controlled for multiple variables (age, race, family history of cancer, hormonal status such as pre-, peri- and post- hormone replacement or contraceptive use and day of menstrual cycle) as potential confounders by using multiple linear regression models or stratified Mantel–Haenszel chi-square analysis as appropriate.

Also, chi-square test was performed to examine correlation between higher T1/T2 ratio obtained by qMRI and early rapid enhancement after gadolinium administration on conventional MRI.

## Results

**Figure 1.**
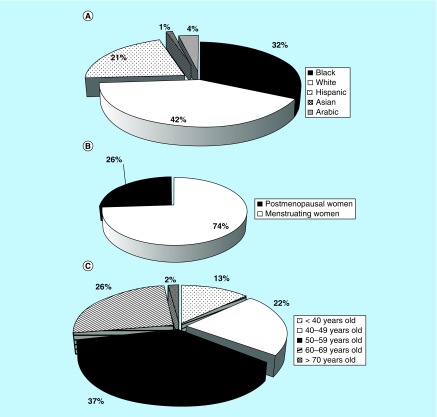
**Demographic variables assessment.** **(A)** Study population assessment by race. **(B)** Menopausal status of study population. **(C)** Age of study subjects. Study population consisted of 32% of black, 42% white, 21% Hispanic 1% Asian and 4 % Arabic women **(A).** Seventy four percent of women that were included in this study were still menstruating and 26% were postmenopausal **(B).** Thirteen percent of women were less than 40 years old, 59% of subjects were older than 40 and 28% were older than 60 years **(C).**

**Figure 2.**
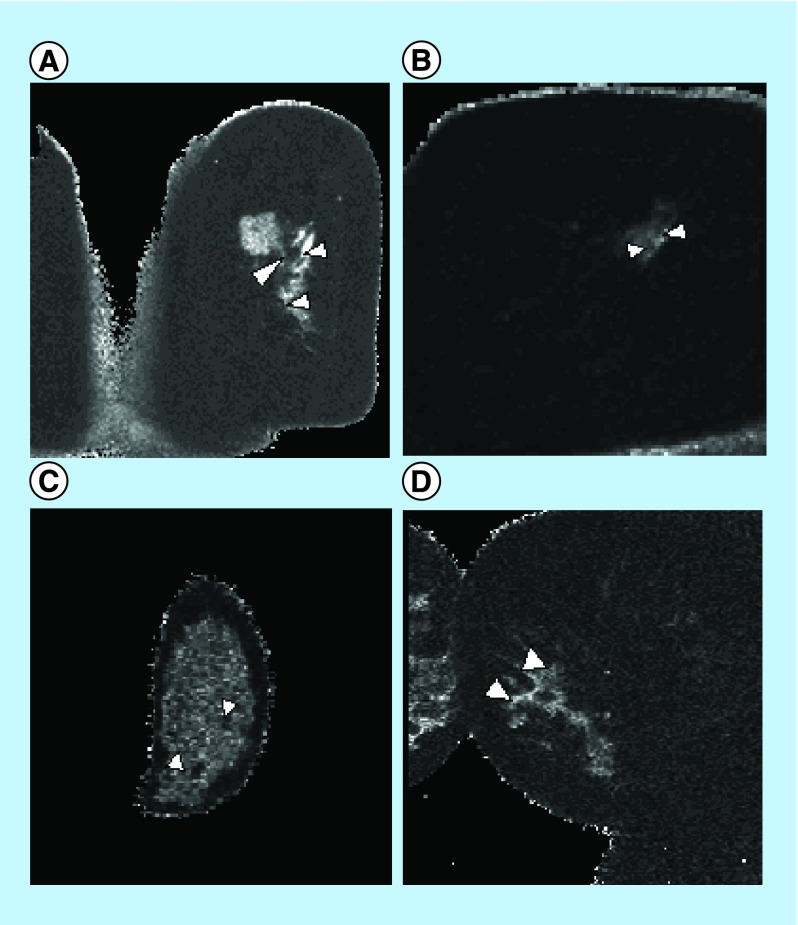
**T1/T2 ratio qMRI maps of analyzed lesions.** Representative T1/T2 ratio qMRI maps demonstrated distinctive appearance that corresponded with pathology findings. White arrowheads point to pathology in the breast tissue. **(A)** Invasive ductal carcinoma in situ (DCIS), Stage 1. Several ring-enhancing lesions around lumpectomy site in right breast may represent residual tumor. **(B)** Invasive lobular carcinoma, Stage 2A status post-lumpectomy, radiotherapy, current Tamoxifen therapy. **(C)** Fibroadenoma, benign. White arrowheads indicated benign lesions in the breast tissue. **(D)** Invasive ductal carcinoma, Stage 1A, newly diagnosed, no prior treatment.

The qMRI analysis was successfully performed in n = 69 subjects with 69 lesions. The study population demographic variables are presented in Figure 1A–C. All 69 analyzed lesions were categorized as benign or malignant based on pathology findings obtained by image-guided or open surgical biopsies. Representative T1/T2 ratio maps with the positioning of rectangular ROIs over lesions are shown in Figure 2.

**Figure 3.**
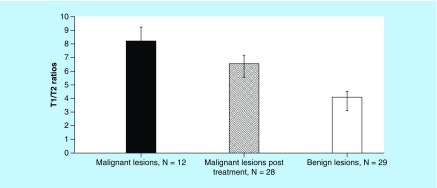
**T1/T2 ratios obtained by qMRI for different breast lesions.** The mean scores obtained by descriptive statistical analysis were 8.20 ±1.05 for Group 1 (n = 12, primary cancer), 6.55 ± 0.63 for Group 2 (n = 28, cancer post-treatment with chemotherapy and/or radiation) and 4.1 ± 0.43 for Group 3 (n = 29, benign lesions included 12 subjects with cysts and 17 with fibroadenoma), respectively.

Descriptive statistical analysis showed different means for the three groups that were analyzed. The T1/T2 ratio means were 8.20 ± 1.05 (n = 12) for biopsy-proven malignant lesions, 6.55 ± 0.63 (n = 28) for malignant lesions that were treated prior to qMRI with chemotherapy and/or radiation and 4.1 ± 0.43 (n = 29) for benign lesion as shown in Figure 3.

**Figure 4.**
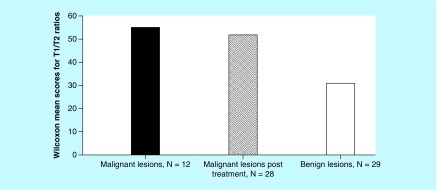
**The mean values of Wilcoxon scores, correlating T1/T2 ratio and the grade of tumor by histopathology, obtained by non-parametric analysis.** These scores were 54.92 for Group 1 (n = 12, primary cancer), 51.82 for Group 2 (n = 28, cancer post-treatment with chemotherapy and/or radiation) and 30.79 for Group 3 (n = 29, benign lesions included 12 subjects with cysts and 17 with fibroadenoma), respectively.

Nonparametric analysis correlating T1/T2 ratio and the tumor grade was performed. The Wilcoxon scores for T1/T2 ratio were assigned based on rank sums that were performed for each group. The mean scores were 54.92 for Group 1 (n = 12, primary cancer), 51.82 for Group 2 (n = 28, cancer post-treatment with chemotherapy and/or radiation) and 30.79 for Group 3 (n = 29, benign lesions included 12 subjects with cysts and 17 with fibroadenoma) respectively. The differences found among these three groups regarding T1/T2 ratio were statistically significant (p = 0.0002) as shown in Figure 4.

These results were reflective of stratification that was performed during data analysis and adjustments were made for confounding variables such as age, race, postmenopausal status, hormonal contraceptives use and personal and family cancer history. Specifically, we controlled these confounding variables by using multiple linear regression models or stratified Mantel–Haenszel Chi-square analysis as appropriate.

Chi-square test showed a strong correlation between higher T1/T2 ratio and a primary cancer group (Group 1) compared with other groups, and the results were statistically significant (p = 0.0335). In addition, the higher stage of cancer determined by histopathology analysis was also strongly associated with higher T1/T2 ratio (p = 0.0093).

**Figure 5.**
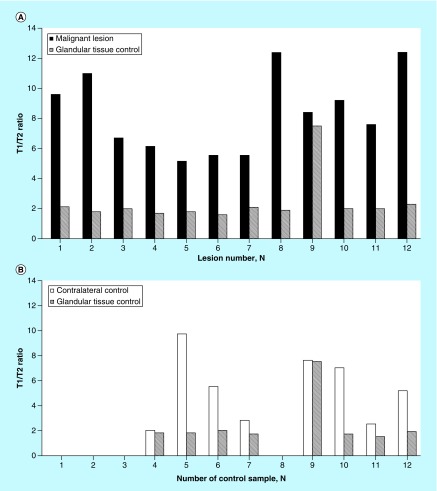

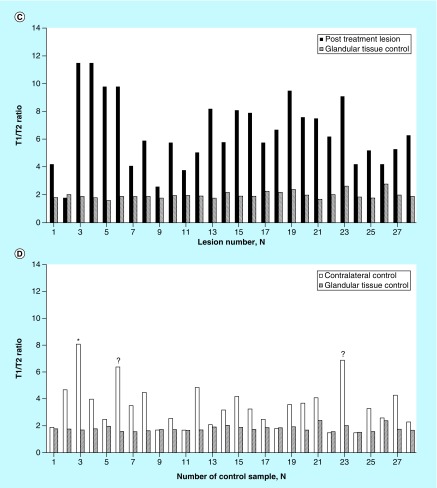

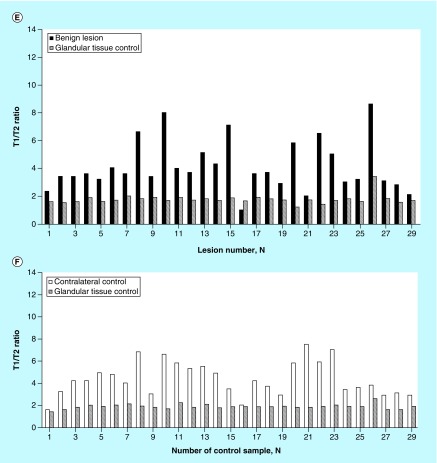
**Contralateral breast and glandular control tissue assessments in primary cancer (A & B), post-treatment group (C & D) and benign lesions (E & F) by qMRI.** In primary cancer group fewer subjects were assessed for contralateral breast since in 4 subjects (samples 1, 2, 3, and 8) only one breast was scanned by MRI due to clinical indication and/or prior mastectomy (n = 8) **(B).** Elevated T1/T2 ratios were detected in three subjects (samples 3 – known bilateral malignancy, 6 and 23 – newly detected) of contralateral breast in post-treatment group (Figure 5D, labelled respectively by “*”, “?”), both were confirmed for malignancy by histopathology analysis.

We analyzed contralateral breast and assessed T1/T2 ratios of glandular tissue as a control sample in all study groups (Figure 5A–F). Since all the measurements were performed post-gadolinium administration, we wanted to be certain that the observed differences in T1/T2 ratio attained by qMRI in examined groups were attributed to pathological changes in the breast tissue and/or in response to treatment. The distribution of T1/T2 ratios for cancer group with corresponding glandular controls and contralateral breast assessments are presented in Figure 5A & B.

The T1/T2 ratio means were 8.20 ± 1.05 (n = 12) for biopsy-proven malignant lesions as compared with glandular tissue control in the same breast of 2.30 ± 0.62 (n = 12) (Figure 5A). According to performed Student’s *t*-test statistical analysis, the differences in T1/T2 ratios observed between malignant and glandular tissues were highly statistically significant (p = 3.06652 × 10^-6^). Fewer subjects were assessed for contralateral breast; in four subjects only one breast was scanned by MRI due to clinical indication and/or prior mastectomy (n = 8) (Figure 5B). The mean T1/T2 ratio for contralateral breast was 5.18 ± 2.74 (n = 8) and 2.39 ± 1.03 (n = 8) for glandular tissue control sample of corresponding breast.

Furthermore, T1/T2 ratio means were 6.55 ± 0.63 (n = 28) for malignant lesions that were treated prior to qMRI with chemotherapy and/or radiation as compared with glandular tissue control in the same breast of 2.99 ± 0.26 (n = 28) (Figure 5C & D). Observed differences in T1/T2 ratios between malignant lesions post-treatment and glandular tissue control were statistically significant (p = 3.5385 × 10^-13^). In post-treatment group, the mean T1/T2 ratio for contralateral breast of was 3.48 ± 0.82 (n = 28) and 1.82 ± 0.22 (n = 28) for glandular tissue control sample of corresponding breast.

Distribution of T1/T2 ratios for benign lesions is shown in Figure 5E & F. The mean T1/T2 ratios were 4.1 ± 0.43 (n = 29) for benign lesion and 1.76 ± 0.35 (n = 29) for glandular tissue control, respectively. The difference in T1/T2 values found between benign lesions and control were statistically significant (p = 6.02714 × 10^-9^). In this group, the mean T1/T2 ratio for contralateral breast was 4.38 ± 1.52 (n = 29) and 1.87 ± 0.21 (n = 29) for glandular tissue control sample of corresponding breast (detailed distribution of T1/T2 ratios is shown in Figure 5E).

Overall, elevated T1/T2 ratios detected in primary cancer and post-treatment group were consistently higher than the ones observed in glandular control tissues, indicating that differences in T1/T2 ratio between examined groups, obtained post-gadolinium administration, were attributed to pathological changes in ductal or lobular breast tissues. In addition, elevated T1/T2 ratios were detected in two samples of contralateral breast in post-treatment group (Figure 5D), both were confirmed for malignancy by histopathology analysis.

In order to explore potential complimentary use of qMRI and conventional breast MRI, we examined the relationship between findings of those two breast imaging techniques. Forty images with confirmed malignancy by histopathology analysis (primary cancer n = 12, post-treatment cancer n = 28) were analyzed by experienced breast imaging radiologist who was blinded to pathology results. We observed that early rapid enhancement after gadolinium administration on conventional MRI did correlate with elevated T1/T2 ratio (p = 0.0007).

## Discussion

We applied qMRI relaxometry of T1/T2 ratio to study breast lesions with different histopathology. This technique allowed mapping the longitudinal-to-transverse relaxation times ratio T1/T2 of benign and malignant breast lesions. It provided a quantitative result and produced high quality image on T1, T2 and T1/T2 maps, as tested on 69 female subjects with 69 known or suspected lesions. Histopathologic analysis was performed for all lesions per standard of care. We found that there was a statistically significant difference in the T1/T2 ratios of benign and malignant breast lesions.

Furthermore, we found statistically significant correlation between higher T1/T2 ratio and higher stage of breast cancer determined by histopathology. Since histopathology is the current standard for diagnosis of breast cancer, this correlation is an important observation as this new qMRI technique may have a role in pre-operative counseling of patients with a new diagnosis of breast cancer.

In addition, early rapid enhancement after gadolinium administration on conventional MRI did correlate with higher T1/T2 ratio obtained by qMRI (p = 0.0007). Therefore, there is a potential for complementary use of those two breast imaging techniques in the future.

Over the past 10 years, MRI of the breast has gained increasing importance in the field of breast imaging. A variety of MRI-based techniques are currently utilized, as summarized below.

First, scientific evidence provided by a considerable number of large-scale trials had confirmed that quantitative measures obtained by MRI using apparent diffusion coefficient as a technique for breast imaging demonstrated unsurpassed sensitivity for primary and recurrent breast cancer [13–15].

Second, Eliata *et al.* had used in multicenter trials a quantitative dynamic contrast enhanced T1 relaxometry [21]. They reported a high sensitivity (around 95%) but lower specificity that varied in a wide range (from 45 to 90%) for characterization of breast tumors [20].

In addition, some scientists utilized dynamic T1-weighted [13,22] and T2-weighted [23,24] MRI imaging for diagnostic assessments of breast tissue or for monitoring response of primary breast cancer to chemotherapy. It was shown that in dynamic breast MR imaging, invasive breast cancers were detectable due to their strong enhancement (signal intensity increase on T1-weighted images) that peaked early after contrast material injection [13,22].

It was reported by Kuhl *et al.* that in a contrast-enhancing breast lesion, careful analysis of T2-weighted TSE images can improve differential diagnosis [23]. It was possible to distinguish between fibroadenomas and breast cancers on those T2-weighted images [23]. However, results for specificity and sensitivity varied significantly between young and older (over 50 years old) patients [23]. Therefore, the accuracy of this breast imaging technique is dependent on age [23]. Bartella *et al.* reported that substantial percentage (24%) of invasive cancers had high or intermediate T2 signal [24].

In summary, it still remains challenging to discriminate malignant lesions from normal or benign structures with MRI. Although listed above techniques provide useful tools for breast cancer diagnosis with high sensitivity (90–95%), a highly specific imaging biomarker is needed [13–24].

Early work by Medina *et al.* used T1 and T2 relaxation times to investigate the ability of NMR spectroscopy to distinguish normal, diseased non-neoplastic and neoplastic human breast tissues [17]. The results indicated that NMR relaxation times could distinguish between the mean values of breast neoplasms and other diseased or normal tissues, with p-values <0.001 [17]. Given a single sample, the probability of classifying it as non-neoplastic or carcinoma could be accomplished with 85% confidence. Medina *et al.* emphasized that for human breast tissues, the relaxation time T2 may be more discriminating than T1 [17]. Medina *et al.* suggested that the T1/T2 ratio could be useful for characterizing breast dysplasias and neoplasms. Furthermore, this T1/T2 ratio has also been found useful for discriminating benign from malignant liver lesions by Farraher *et al.* [18].

In our study, we found strong correlation between findings of contrast enhanced MRI and our qMRI results. In addition, the qMRI offered a quantitative approach to the assessment of breast lesions, whereas conventional MRI only provides qualitative information. Furthermore, elevated T1/T2 ratios have been observed in hepatocellular carcinomas previously by Farraher *et al.* [18].

Also, qMRI of T1/T2 could be utilized in questionable cases for evaluation of patients with abnormal hyperplasia to non-invasive and low-grade invasive carcinoma in order to distinguish better among these borderline conditions. It may be potentially useful technique in women with questionable mammographic and clinical findings, in patients with previous breast surgery to better distinguish post-surgical scar from recurrent carcinoma and also in women with very dense breasts or to evaluate small lesions.

Further prospective, larger studies are needed that will enable use of receiver operator curves, obtained by qMRI technique for breast tissues. These studies will provide additional data for sensitivity and specificity analysis to establish diagnostic value of qMRI for characterization of breast lesions.

### Limitations of the study

One potential limitation stems from acquiring images after gadolinium injection, which affects the relaxation times. All mixed-TSE scans were run at approximately 5–6 min after the gadolinium was initially injected and this time interval was kept constant among subjects in order to minimize potential relaxometric inconsistencies among subjects. Because of the long duration of the MRI examination and in consideration of research subjects comfort, it was decided for this pilot study to run the research scan last, so to give subjects a possibility to opt in/out in case of high discomfort.

The encouraging diagnostic findings of this post-gadolinium study could serve as a motivation to explore further the value of the T1/T2 ratio pre-contrast administration. Further pre-gadolinium administration studies will be necessary to fully establish the clinical value of this qMRI parameter. Nevertheless, from a pragmatic point of view, the post-gadolinium T1/T2 ratio measured consistently 5–6 min post-contrast injection in all subjects, appears to be a promising diagnostic tool for evaluation of breast disease and could potentially lead to the reduction of breast biopsies.

Generalizability of the present results is limited to post-menopausal women and women who are between days 5 and 10 of menstrual cycle, since we excluded women that were in a different part of their menstrual cycle in order to avoid hormonal fluctuations and decrease variability of MRI results. In addition, the Asian population was under-represented (only 2% of the studied cohort). Therefore, the results of the study could only be generalized to Caucasian, Black and Hispanic women. Additional studies are needed to assess this technique in other ethnic groups.

Since this was a cross-sectional study, the post-treatment cancer group was not homogeneous. Specifically, based on inherited limitation of the study design, we could not avoid including subjects at the different time points of their treatment (e.g., variable cycles of chemotherapy were performed by individual subjects prior to qMRI assessment) and/or subjects on different treatment regimens (e.g., different combinations of chemotherapy and/or radiotherapy).

Also, in the current study, different histopathological types of breast malignancy (e.g., ductal and lobular carcinoma) were combined due to the relatively small numbers for lobular carcinoma. We only examined two subjects with lobular carcinoma out of 69 assessed subjects in which majority of cases were with ductal carcinoma. It did not permit an analysis of whether invasive ductal or lobular cancers exhibit different quantitative properties on qMRI. This issue needs to be explored in future studies.

## Conclusion & future perspective

In summary, we found a significant difference in the T1/T2 ratios of benign and malignant breast lesions. In addition, a significant correlation between elevated T1/T2 ratios and more advanced stage of breast cancer confirmed by histopathology was observed.

Combining quantitative information of different tissue properties such as T1/T2 ratio may provide a useful additional diagnostic tool for breast cancer. This could potentially translate into establishing imaging biomarkers of breast cancer and reduction in the number of benign breast biopsies.

This qMRI approach may provide valuable complementary information to established breast imaging sequence which can possibly translate into improved diagnosis of breast cancer.

**Executive summary**

**Background**

- Breast cancer is one of the most common cancers among women. Early detection is important for successful intervention and improved prognosis. Advances in the treatment and outcome of patients with breast cancer require the development of diagnostic tools with highy positive predictive value.
- Imaging modalities such as mammography, ultrasonography and MRI are routinely used to identify and characterize breast lesions. MRI is recognized as a supplement to mammography in the visualization and characterization of breast lesions. The purpose of this work was to measure the T1/T2 ratio of a variety of breast lesions and to correlate with histopathological findings with the goal of increasing the diagnostic power of MRI.

**Materials & methods**

- We performed a cross-sectional study of subjects scheduled to undergo conventional MRI.
- Participants were asked to undergo additional 5–12 min of qMRI sequence after conventional MRI is performed. Post-menopausal status, age, hormone-replacement therapy and contraceptives use were recorded to perform stratification at data analysis. All images were obtained between days 5 and 10 of menstrual cycle in order to have consistency in MRI data.
- Images obtained with the mixed-turbo spin echo pulse sequence in the coronal plane of the entire breast were post-processed for generating T1 and T2 distributions, which were spatially co-registered. These were used as input for a T1/T2 ratio algorithm to distinguish between benign and malignant breast lesions.
- Descriptive statistical analysis and nonparametric analysis correlating T1/T2 ratio and the tumor grade was performed. The Wilcoxon scores for T1/T2 ratio were assigned based on rank sums that were performed for each group.
- We analyzed contralateral breast by qMRI and assessed T1/T2 ratios of glandular tissue as a control sample in all study groups. Correlations between T1/T2 ratios were made with histopathology findings and conventional MRI results.

**Results**

- Elevated T1/T2 means of 8.20 ± 1.05 (n = 12) were observed for biopsy-proven malignant lesions and 6.55 ± 0.63 (n = 28) for malignant lesions that were treated prior to qMRI with chemotherapy and/or radiation as compared with 4.1 ± 0.43 (n = 29) for benign lesions.
- The T1/T2 ratios obtained by qMRI strongly correlate with histopathological findings. The nonparametric analysis revealed correlation between elevated T1/T2 ratio and more advanced disease stage.
- Overall, elevated T1/T2 ratios detected in primary cancer and post-treatment group were consistently higher than the ones observed in glandular control tissues, indicating that differences in T1/T2 ratio between examined groups, obtained post-gadolinium administration, were attributed to pathological changes in ductal or lobular breast tissues.
- We observed that early rapid enhancement after gadolinium administration on conventional MRI did correlate with elevated T1/T2 ratio (p = 0.0007).

**Discussion**

- We applied quantitative MRI relaxometry of T1/T2 ratio to study breast lesions with different histopathology. It provided a quantitative result and it produced high quality image on T1, T2 and T1/T2 maps, as tested on 69 female subjects with 69 known or suspected lesions.
- We found statistically significant correlation between higher T1/T2 ratio and higher stage of breast cancer determined by histopathology, which is currently a standard for diagnosis of breast cancer.
- In addition, early rapid enhancement after gadolinium administration on conventional MRI did correlate with higher T1/T2 ratio obtained by qMRI (p = 0.0007). Therefore, there is a potential for complementary use of those two breast imaging techniques in future.

**Conclusion & future perspective**

- Quantitative MRI approach may provide valuable complementary information on different tissue properties to established breast imaging sequence as additional diagnostic tool which can possibly translate into improved diagnosis of breast cancer.
